# The colder is warmer? A pre-study for wear trials of a reference clothing ensemble for EN 342 and EN 14058 for thermal manikin calibration

**DOI:** 10.1186/2046-7648-4-S1-A161

**Published:** 2015-09-14

**Authors:** Kalev Kuklane, Muhammad Salman Butt, Amitava Halder, Chuansi Gao

**Affiliations:** 1 Division of Ergonomics and Aerosol Technology, Department of Design Sciences, Faculty of Engineering, Lund University, Lund, Sweden

## Introduction

Two sets of calibration ensembles for thermal manikins are available at present for EN 342 (and 14058). These 2 sets are not fully enough as: a) statistically, 2 points give an ideal regression line that does not need to be correct for extended range; b) they do cover an insulation range of only EN 14058 and the lower end of EN 342 - the insulation range of very cold and extremely cold exposures (below -5 °C), that is the main concern of EN 342, is not covered. The aims of the pre-test were to: a) settle the preliminary test conditions; b) test the equipment in extreme cold (down to -40 °C); c) suggest a metabolic rate for testing.

## Methods

Ensemble C of Subzero project (cold store ensemble, SZC, [1]) was selected for testing. Three subjects participated in the pre-tests for wear trials. They were walking on a treadmill at 3.5 km/h. In Part 1 S1 was walking at +20 °C (with and without winter clothes) for 30 minutes, and at -2 and -36 °C, as well as S2 at -38 °C (with SZC) for 90 minutes. In Part 2 S3 was walking for 20-30 minutes at +10 (with and without winter clothes), -10, -20 and -34 °C (with SZC). Bending stiffness (Pierce's test) of the sections of clothing outer layer was measured as a function of ambient temperature (used in Part 2). Different instruments and methods were used to analyse metabolic heat production from oxygen consumption (VO_2_): in Part 1 Metamax I and Metamax II and in Part 2 Metamax I and Douglas bag method. Other recorded parameters were body weight loss, heart rate, thermal (range from +4 to -4) and pain sensation (range from 0 to 3).

## Results

Part 1: Participants were able to walk in extreme cold for 1.5 hours. Toes started feeling cold after 45 min, pain was reported at around 50-55 min, and discomfort grew with time to the end of the exposure. Estimated metabolic rate (M) according to Givoni and Goldman [2] was 140 W/m^2^. Estimated M from ISO 8996 for walking without load on level and even surface (mean of 3 and 4 km/h) was 153 W/m^2^. M of S1 and S3 without winter clothes was around 160 W/m^2^, M for them with SZC under warm and cold conditions about 180 W/m^2^, and below -30 °C for all about 200 W/m^2^. Based on Part 2 it was ruled out that the treadmill speed could be affected by cold by counting belt rotations. The effect of cold on measured instrument values was ruled out, too, by comparing the results of different equipment that measured VO_2_.

## Discussion

The metabolic rate seemed to increase at higher step rate (results from 1 subject only!). Would higher step rate strengthen clothing stiffness effect? It may be possible that the garment gets stiffer in cold and thus increases motion resistance. Previous research suggests that clothing stiffness may affect metabolic rate by 10 % in addition to the protective clothing own effect (up to about 20 %, [3]).

## Conclusion

The extreme cold may affect the instruments and measurement accuracy. However, these can be avoided by warming the sampling lines, removing ice if needed or using the Douglas bags. Cadence (step/min) may affect the results. If comparing the subjects and the manikin then the subjects should be requested to walk at the same frequency (45 double steps/min). Cold affects clothing stiffness. Most probably the effect is material dependent. Although the planned study was not performed, these pre-study results do define test parameters, and would provide possibility to run comparative tests with short notice.
